# Calreticulin Is a Negative Regulator of Bronchial Smooth Muscle Cell Proliferation

**DOI:** 10.1155/2012/783290

**Published:** 2012-02-21

**Authors:** Nicola Miglino, Michael Roth, Didier Lardinois, Michael Tamm, Peter Borger

**Affiliations:** Departments of Biomedicine, Pulmonary Cell Research, and Thorax Surgery, University Hospital Basel, Hebelstrasse 20, 4031 Basel, Switzerland

## Abstract

*Background*. Calreticulin controls the C/EBP**α**p42/p30 at the translational level trough a cis-regulatory CNG rich loop in the CEBPA mRNA. We determined the effects of steroids and long-acting beta-agonists on the p42/p30 ratio and on calreticulin expression in primary human bronchial smooth muscle (BSM) cells. *Methods*. The effects of budesonide (10^−8^ M) and formoterol (10^−8^ M) were studied in BSM cells pre-treated with siRNA targeting calreticulin. The expression of C/EBP**α** and calreticulin was determined by immuno-blotting. Automated cell counts were performed to measure proliferation. *Results*. All tested BSM cell lines (*n* = 5) expressed C/EBP**α** and calreticulin. In the presence of 5% FBS, the p42/p30 ratio significantly decreased (*n* = 3, *P* < 0.05) and coincided with BSM cell proliferation. High levels of calreticulin were associated with a decreased p42/p30 isoform ratio. FBS induced the expression of calreticulin (*n* = 3, *P* < 0.05), which was further increased by formoterol. siRNA targeting calreticulin increased the p42/p30 ratio in non-stimulated BSM cells and significantly inhibited the proliferation of PDGF-BB-stimulated BSM cells (*n* = 5, *P* < 0.05). Neither budesonide nor formoterol restored the p42 isoform expression. *Conclusions*. Our data show calreticulin is a negative regulator of C/EBP**α** protein expression in BSM cells. Modulation of calreticulin levels may provide a novel target to reduce BSM remodeling.

## 1. Introduction

An important feature of asthma pathology is airway wall remodeling, characterized by a thickened basement membrane and an increase with respect to the bulk of the bronchial smooth muscle (BSM) cells [1–3]. Earlier we showed that the increased proliferation rate of asthmatic BSM cells was normalized after the introduction of an expression vector for full length C/EBP*α* mRNA [4]. We have further provided data showing a disease specific expression of C/EBP-isoforms in asthma and COPD [5].

C/EBP*α* can be expressed as full length and truncated protein isoforms, commonly referred to as p42 and p30. The full length C/EBP*α* (p42) functions as a proliferation inhibitor, whereas the truncated C/EBP*α* (p30) does not have this effect [6, 7]. A decreased p42/p30 ratio may therefore render BSM cells a growth advantage and result in thicker layers of muscles around the airways as observed in the lungs of asthma patients.

The standard therapy for asthma consists of drugs that reduce airway inflammation (predominantly glucocorticoids) and induce relaxation of the smooth muscles (predominantly *β*_2_-agonists). We have earlier shown the molecular biological basis of the interaction of both classes of drugs, which involved the formation of a complex consisting of the glucocorticoid receptor and C/EBP*α* [8–11]. This complex is able to activate the cell cycle inhibitor p21^cip1/waf1^ [9, 10], thus demonstrating an interactive negative regulatory network for cell proliferation. The observed diminished expression of C/EBP*α* in BSM cells of asthma patients is mainly due to posttranscriptional regulation affecting the translation of the *CEBPA* mRNA [12, 13].

**Figure 1:**
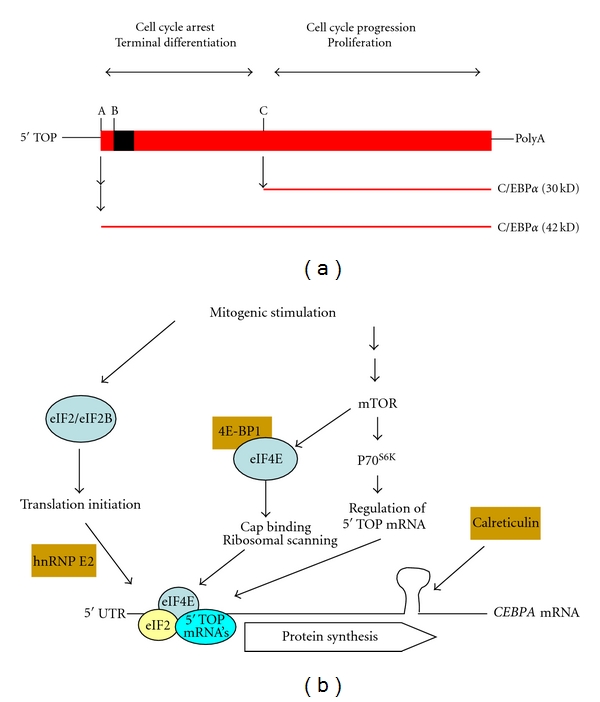
(a) Simplified scheme of the *CEBPA* mRNA. Due to alternative translation start sites (A, B, and C), full length (p42) and truncated (p30) C/EBP*α* proteins with distinct functions can be formed. Start site B is out of frame and determines whether A or B is accessible for translation, hence producing either p42 or p30 C/EBP*α* proteins. (b) Schematic representation showing three important signaling pathways for the translation control of* CEBPA* messenger RNA: (1) the pathway leading to activation of the eukaryotic initiation factors eIF2 and eIF2B, which is counteracted by hnRNPE2, (2) the pathway of mTOR and eukaryotic initiation factor 4E (eIF4E), which is inhibited by 4E-BP1, and (3) the pathway leading to calreticulin (CRT) expression, a protein that binds to a double-stranded RNA loop and prevents the translation of full length C/EBP*α* proteins. Abbreviations: 5′ TOP: 5′ tract of pyrimidine; mTOR: mammalian target of rapamycin.

In general, two mechanisms can be involved in translation control: “global” and “selective”. Global control acts on all mRNAs in a nonspecific manner, whereas selective translation regulation targets a specific subset of mRNAs. These specific mRNAs often have *cis*-regulatory sequences that sense subtle changes in the activity of the translation machinery or form loops that affect the accessibility of the appropriate translation start sites. It is now well documented that the *CEBPA *mRNA can be expressed as a full length protein (p42) or a truncated form (p30) [6, 7, 14–18]. The p42/p30 ratio is predominantly controlled at the translational level [15–18]. Full lengths (p42) and truncated (p30) C/EBP*α* proteins are generated from one single 5′ tract of pyrimidine (5′ TOP) *CEBPA* mRNA (Figure 1(a)). Three important signaling pathways regulate the translation of 5′ TOP messengers (Figure 1(b)). The first is the ubiquitous eukaryotic initiation factor 2 (eIF2). The second is leading to activation of mammalian target of rapamycin (mTOR) and subsequent activation of eukaryotic initiation factor 4E (eIF4E). These pathways are stringently controlled by specific inhibitory proteins, including hnRNPE2, which interferes with translation initiation, and 4E-BP1, a protein that prevents ribosomal scanning [19]. The third level of control of *CEBPA* mRNA translation is found in a *cis*-regulatory double-stranded RNA loop, which provides a docking site for calreticulin. When calreticulin is bound to this sequence, translation of the full length C/EBP*α* (p42) is reduced [20].

Here, we isolated and maintained primary human BSM cells and studied the involvement of calreticulin in the regulation of *CEBPA* mRNA translation and whether budesonide and formoterol are able to modulate the p42/p30 ratio.

## 2. Material and Methods

### 2.1. Tissue Specimens and Cell Cultures

 Lung tissue specimens were obtained from the Department of Internal Medicine, Pneumology, and the Department of Thorax Surgery, University Hospital Basel, Switzerland, with the approval of the local Ethical Committee and written consent of all patients. BSM cells were established as previously described [10] and grown in RPMI 1640 (Lonza, Basel, Switzerland) supplemented with 5% fetal bovine serum (FBS), 8 mM L-glutamine, 20 mM HEPES, and 1% MEM vitamin mix (Gibco, Paisley, UK). Neither antibiotics nor antimycotics were added at any time.

### 2.2. Cell Treatment and Drugs

 Confluent BSM cells were cultured for 24 hours in the presence or absence of 5% FBS and grown in the presence of an optimal concentration [4, 8–10] of budesonide (10^−8 ^M), formoterol (10^−8 ^M), or a combination of both drugs for 24 and 96 hours.

### 2.3. Small Inhibitory RNA (siRNA) Treatment

Transfection with siRNA for calreticulin or negative control (Ambion, Austin, USA) was performed according manufacturer protocol. Cells (70% confluence) were plated into 6 well plates and transiently transfected with siRNA (50 nM) for 6 hours. Thereafter, fresh RPMI was added for 24 hours. Then, cells were cultured in presence or absence of budesonide (10^−8 ^M) or formoterol (10^−8 ^M). Cell lysates were collected after 24 hours and prepared for immuneblot analysis.

### 2.4. Protein Isolation and Analysis by Immunoblot

 Cellular proteins were isolated from confluent cells by dissociation in lysis buffer (62,5 mM Tris-HCl (pH 6.8), 2% SDS, 2% *β*-mercaptoethanol, 10% glycerol) and denaturation in sample buffer (3x Laemmli buffer with *β*-mercapto-ethanol) and boiling for 5 min. Equal protein amounts were loaded onto a 4–12% PAGE-gel (Pierce Biotech, Thermo Fisher Scientific, Rockford, IL, USA) and were size fractionated by electrophoresis (1 hr, 100V, open A). The gel was sandwiched between two nitrocellulose membranes (Biorad, Reinach, Switzerland), and proteins were transferred (transfer buffer: 0.05 M NaCl, 2 mM Na-EDTA, 0.1 mM DTT, 10 mM, Tris HCl (pH 7.5)) overnight (50°C). Protein transfer and equal loading were confirmed by Ponceau's staining. The membranes were blocked (10 min) in 3% bovine serum albumin (Roche, Rotkreuz, Switzerland) in 1x phosphate buffered saline with 0.05% Tween-20 (PBST). The membranes were incubated (1 hour) at room temperature (RT) with one of the antibodies to C/EBP*α* (Santa Cruz Biotech, Santa Cruz, USA) and calreticulin (Santa Cruz Biotech). Membranes were then washed (3 × 5 min) and incubated (1 hour, RT) with horseradish labeled species-specific antibodies (Santa Cruz Biotech). The membranes were washed (3 × 5 min) with and incubated (5 min) with ECL-substrate (Pierce), and protein bands were visualized on X-ray films (Fuji Film, Medical X-ray film, Luzern, Switzerland). Protein bands were semiquantified by an image analysis system (ImageJ). Protein expression was normalized to *α*-tubulin as internal control. The presented p42/p30 ratios were calculated from normalized densitometry data.

### 2.5. Proliferation

 BSM cells were plated in a 24 wells plate at a density of 10^4^ cells/well. Next cells were grown for 24 hours in the presence of FBS (5%), before being serum starved for 24 hours. Then, cells were incubated in absence or presence of FBS (5%) for 96 hours. After trypsinization cells, were counted manually and/or by using an automated particle counter (Coulter).

### 2.6. Statistics

 Cytokine and proliferation data are presented as mean ± SEM, immunoblot analysis is shown as mean ± SEM after densitometric image analysis (ImageJ software, National Institute of Mental Health, Bethesda, MD, USA) of independent experiments. Paired/unpaired Student's *t*-test was performed, and *P*  values <0.05 were considered significant.

## 3. Results

### 3.1. Effects of FBS, Budesonide, and Formoterol on p42/30 Ratios

**Figure 2:**
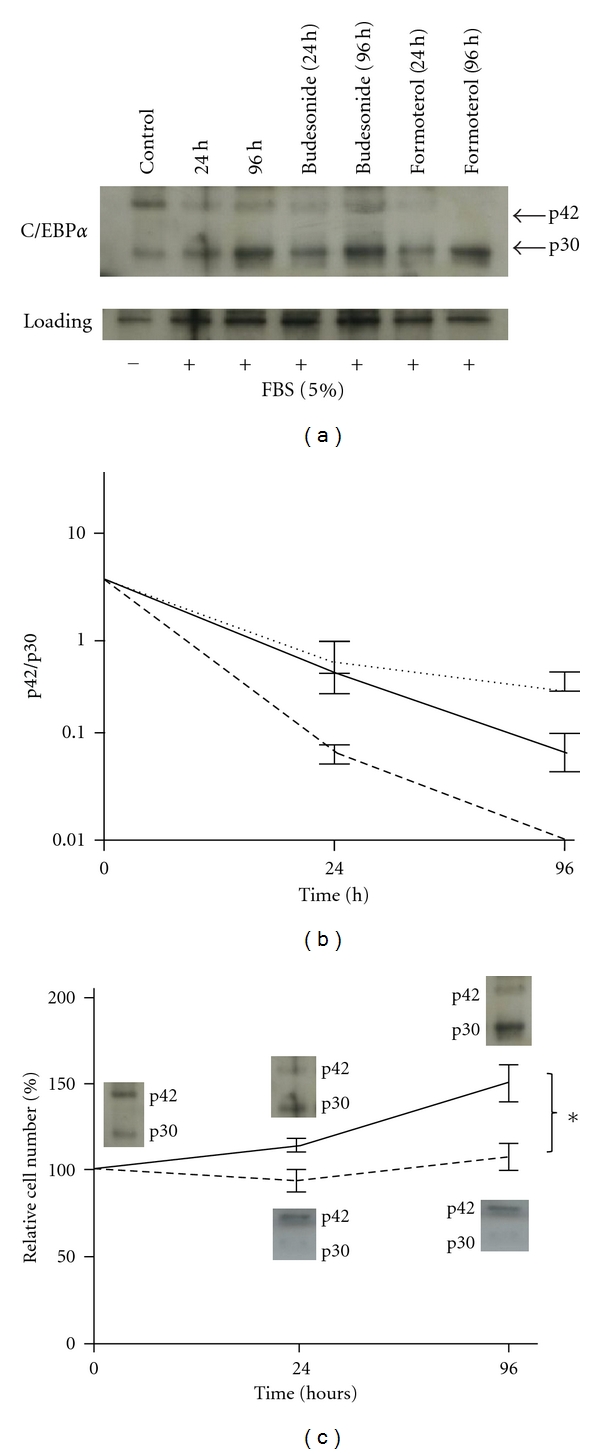
(a) Representative immunoblot analysis demonstrating the modulatory effect of asthma drugs on the C/EBP*α* (p42) and C/EBP*α* (p30) expression pattern in human BSM cells. BSM cells were untreated (control) or incubated with 5% FBS alone, and in the presence of budesonide (10^−8 ^M) or formoterol (10^−8 ^M) for 24 and 96 hours. Similar data were obtained in two additional cell lines. (b) C/EBP*α* p42/p30 ratios (mean ± SEM; *n* = 3) in BSM cells cultured for 0, 24, and 96 hours with 5% FBS (solid line), budesonide (dotted line), or formoterol (dashed line). (c) C/EBP*α* (p42 and p30) expression patterns projected in the proliferation curve of BSM cells. Cells were incubated for 0, 24, and 96 hours in absence (dotted line) or presence of 5% FBS (solid line). Data are expressed as a percentage of the cell number at *t* = 0 (control). *Significant difference between untreated and FBS-stimulated cells (*P* < 0.05; *n* = 4). Photo insets show the corresponding ratio of C/EBP*α* p42 and p30 expression of one representative experiment at *t* = 0 h (control), *t* = 24 h, and *t* = 96 h.

**Figure 3:**
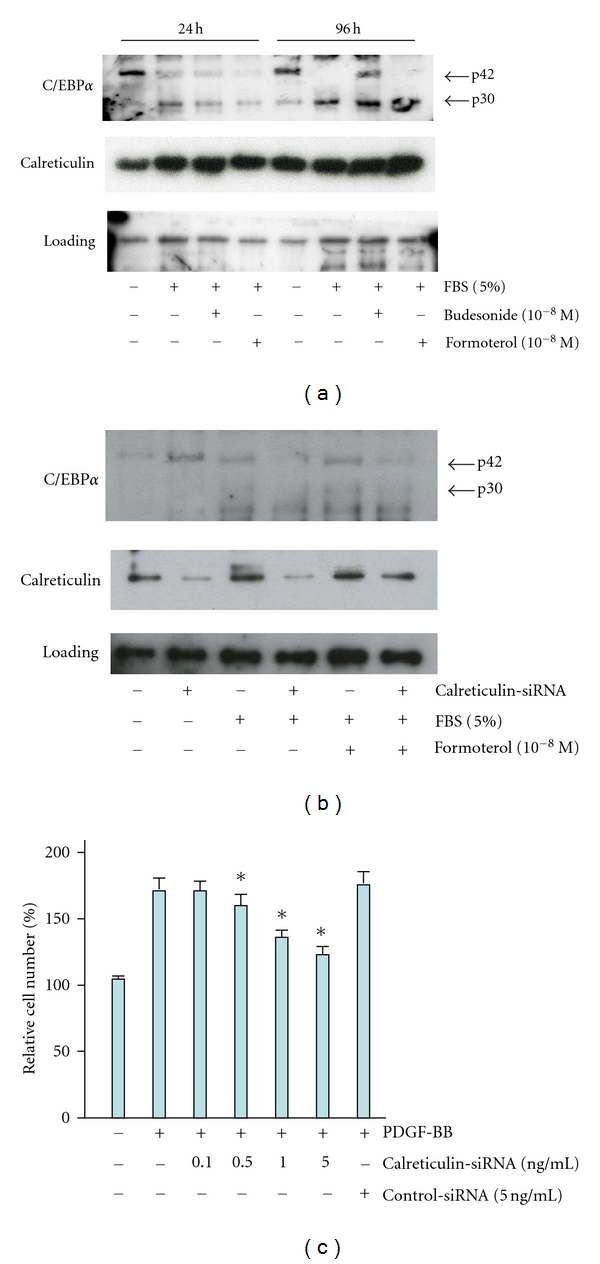
(a) Immunoanalyses demonstrating the effect of asthma drugs on the C/EBP*α* (p42), C/EBP*α* (p30), and calreticulin expression pattern. BSM cells were untreated or incubated with 5% FBS alone, and in the presence of budesonide (10^−8 ^M) or formoterol (10^−8 ^M) for 24 and 96 hours. (b) Immunoanalyses demonstrating C/EBP*α* (p42), C/EBP*α* (p30), and calreticulin expression patterns in BSM cells after transient knockout of the calreticulin by siRNA. BSM cells were untreated or incubated with 5% FBS alone, and in the presence of formoterol (10^−8 ^M) for 24 hours. (c) BSM cell proliferation (presented as relative cell counts) in response to PDGF-BB (5 ng/mL) and the effect of increasing concentrations calreticulin-specific siRNA relative to control siRNA (as indicated). *Significant inhibition relative to PDGF-BB-stimulated cells (*P* < 0.05; *n* = 5).

To determine the effect of FBS, budesonide, and formoterol on p42/p30, BSM cells (*n* = 3) were cultured for 0, 24, and 96 hours in growth medium (5% FBS) supplemented with either budesonide (10^−8 ^M) or formoterol (10^−8 ^M). FBS (5%) significantly reduced p42 C/EBP*α* levels (*n* = 3; *P* < 0.05), both after 24 and 96 hours (Figure 2(a)). Concomitantly, p30 C/EBP*α* levels were significantly increased (*n* = 3; *P* < 0.05) at both time points, resulting in reduced p42/p30 ratios which are presented in Figure 2(b). The addition of formoterol to FBS stimulated BSM cells further reduced the p42/p30 ratio below 0.01, whereas budesonide did not modify the effect of FBS (Figure 2(b)). As shown in Figure 3(c), a reduced p42/p30 ratio coincided with a significantly increased proliferation rate of 5% FBS-stimulated BSM cells relative to nonstimulated cells (*n* = 4; *P* < 0.05).

### 3.2. Calreticulin Levels Coincide with C/EBP p42/p30 Ratios

BSM cells were incubated with FBS (5%) supplemented with budesonide (10^−8 ^M) or formoterol (10^−8 ^M). As demonstrated in Figure 3(a), resting BSM cells expressed low levels of calreticulin, which were significantly upregulated after 24 an 96 hours in the presence of 5% FBS, only. After 96 hours, budesonide (10^−8 ^M) slightly restored the expression of the p42 isoform, but the p42/p30 ratio was unaffected. The expression of calreticulin protein coincided with low levels of C/EBP*α* (p42) and high levels of C/EBP*α* (p30): the p42/p30 ratio significantly decreased (*n* = 3; *P* < 0.05). As shown in Figure 3(b), calreticulin-specific siRNA significantly decreased the expression of calreticulin protein (*n* = 3, *P* < 0.05). The knockdown of calreticulin only increased the expression of C/EBP*α* (p42) in untreated BSM cells. Finally, BSM cells were transfected with increasing concentrations of calreticulin-specific siRNA and incubated in presence and absence of PDGF-BB for 96 hours. As demonstrated in Figure 3(c), siRNA for calreticulin dose-dependently inhibited the proliferation of PDGF-BB-stimulated BSM cells (*n* = 5; *P* < 0.05 for calreticulin-siRNA ranging from 0.1 to 5.0 ng/mL), whereas the control siRNA did not have an effect on PDGF-BB-induced proliferation (*n* = 5; *P* = 0.59).

## 4. Discussion

An increased capacity to proliferate is a key feature of BSM cells obtained from asthma patients and may provide an explanation for the observed increase of BSM bundles surrounding the bronchi of asthma patients [21]. We have extensively explored the role of the *CEBP* transcription factor-family in BSM cell proliferation and concluded that the expression and regulation of C/EBP*α* isoforms may be crucial to understand the proliferation control of BSM cells [4, 5, 12, 13]. In our present study, we demonstrated that normal BSM cells express C/EBP*α* (p42) and C/EBP*α* (p30) isoforms, as well as their specific translation regulator calreticulin. Moreover, we observed a specific relation between these proteins, that is, when calreticulin levels are high, the p42/p30 ratio is small. Furthermore, we showed that in the presence of serum, the p42/p30 ratio significantly decreased. Addition of budesonide, but not formoterol, slightly restored the p42 levels. Restoring p42 levels would theoretically restore the responsiveness of BSM cells to budesonide and/or formoterol, since only full length C/EBP*α* proteins formed a complex with the glucocorticoid receptor to induce the cell cycle inhibitor p21^cip1/waf1^[9, 11]. Both budesonide and formoterol were unable to significantly increase the p42/p30 ratio, however. The incapability of budesonide and formoterol to induce the expression of C/EBP*α* (p42) may explain why the airway remodeling observed in asthma patients is resistant to therapy involving steroids and/or *β*-mimetics [22].

We have earlier reported that an impaired translation-initiation of the *CEBPA* mRNA in BSM cells of asthma patients was associated with the decreased expression of the translation regulator eIF4E [12]. We were unable to detect significant differences with respect to eIF4E levels between house-dust-mite-challenged BSM cells isolated from asthmatic and nonasthmatic subjects, however [13]. Therefore, we proposed that calreticulin, a protein initially identified as an endoplasmic reticulum luminal chaperone that controls the regulation of intracellular Ca^2+^ homeostasis [23], could be pivotal in the downregulation of C/EBP*α* translation and may be one of the key regulators to explain low levels of C/EBP*α* proteins in BSM cells of asthma patients. Binding of calreticulin has been shown to inhibit the translation of the *CEBPA* mRNA, as a result of a direct interaction of calreticulin and the *CEBPA* transcript. As depicted in Figure 1(b), calreticulin binds to a stem loop within the *CEBPA* mRNA, which is formed by internal base-pairing of the GCN repeat motif [24]. When calreticulin is bound to this loop, translation of the full length C/EBP*α* (p42) can no longer be generated and p21^cip1/waf1^ cannot be formed. An inverse relationship of C/EBP*α* and calreticulin had been demonstrated in adipocytes, where calreticulin inhibited adipogenesis by suppressing the expression of C/EBP*α* [25]; an observation that was also reported in acute myeloid leukemia [26]. Here, we demonstrated that the same mechanism operates in normal BSM cells, because a transient suppression of calreticulin by siRNA increased C/EBP*α* (p42) levels in resting BSM cells. It should be noted, however, that in proliferating cells additional mechanisms operate to control C/EBP*α* isoforms [4, 12, 13]. Therefore, the decrease of the C/EBP*α* protein level in BSM cells of asthma patients may only partially be related to increased calreticulin.

Our current data show that in the presence of 5% FBS BSM cells rapidly decreased the p42/p30 ratio. Formoterol was able to even further reduce the p42/p30 values. Here, the p42/p30 value went below 0.01, demonstrating an additive effect relative to FBS alone (Figure 2(b)). This additive effect was not observed with budesonide and indicates that formoterol also activates additional pathways not induced by budesonide and independent of C/EBP*α* (p42). It should be emphasized that both C/EBP*α* (p42) and C/EBP*α* (p30) can bind to the same DNA motifs but that p30 cannot exert the antiproliferative effects of p42. The p42, however, is a direct inhibitor of cell cycle progression.

Finally, we found that the siRNA targeting calreticulin dose-dependently inhibited BSM cells proliferation and restored C/EBP*α* (p42) in nonstimulated BSM cells only. This shows that, although FBS was able to induce calreticulin, it does not exert its effects through an increased expression of calreticulin. Rather, FBS and PDGF may affect the transcription of the gene directly or redirect the translation machinery to alternative start codons present in the *CEBPA* mRNA as described previously [14–16]. Calreticulin levels were slightly increased after 96 hours of treatment with formoterol. The significance of this observation is currently unclear, but may indicate an additional inhibitory effect on proproliferative members of the *CEBP* gene family [24].

Taken together, our current data demonstrate that the translation-controlled C/EBP*α* (p42) and its counterpart (p30) are present in BSM cells. Calreticulin functions as an important control protein for BSM cell proliferation, but largely independent of the transactivating C/EBP*α* protein isoform. However, modulation of calreticulin levels—either (epi) genetically or by administration of specific drugs—may be a novel tool to target remodeling parameters involving BSM cells, both *in vitro* and *in vivo*.
